# Stretchable Textile-Based Membraneless Microfluidic Microalgae–Microbial Solar Cell

**DOI:** 10.3390/mi17050593

**Published:** 2026-05-13

**Authors:** Hui Geon Kong, Yeon Woo Cha, Sang Hyuk Lee, Injun Song, Yoomin Ahn

**Affiliations:** Department of Mechanical Engineering, BK21 FOUR ERICA-ACE Center, Hanyang University, 55 Hanyangdaehak-ro, Sangnok-gu, Ansan 15588, Gyeonggi-do, Republic of Korea

**Keywords:** microalgae biocathode, co-cultured, fabric-based, flexible photosynthetic microbial fuel cell, co-laminar flow, self-sustainable, mediator-less

## Abstract

A textile-based membraneless microfluidic microalgae–microbial solar cell (μmMSC) was developed for low-cost, flexible, and sustainable power generation. Unlike conventional systems, the proposed device utilizes a textile substrate, enabling mechanical flexibility and simplified fabrication. Microfluidic channels were patterned via screen printing using hydrophobic Ecoflex, and conductive electrodes were fabricated using PEDOT:PSS combined with Ag_2_O and carbon nanotubes (MWCNT/SWCNT). At the anode, Synechocystis sp., Bacillus subtilis, and Shewanella oneidensis MR-1 were vertically co-cultured to enhance synergistic bioelectrochemical activity, while Scenedesmus obliquus was employed as a microalgae-based biocathode. Under these conditions, the μmMSC achieved a maximum current density of 144 μA cm^−2^ and a peak power density of 17 μW cm^−2^. These results demonstrate that the proposed textile-based μmMSC provides a promising platform for flexible bio-solar energy systems, with potential for wearable applications, while offering improved sustainability and scalability compared to conventional rigid device.

## 1. Introduction

The wearable device market is expanding rapidly, driven by the advancement of the Fourth Industrial Revolution and the development of Internet of Things (IoT) technologies. The importance of wearable devices is also increasing in smart manufacturing and warehouse applications associated with Industry 4.0 trends [1]. Compared to conventional secondary batteries with limited lifespans, biofuel cells have attracted significant attention as promising self-sustainable power sources due to their low cost, environmental friendliness, and ease of fabrication [2]. Key requirements for wearable power systems include miniaturization, flexibility, and long-term sustainability. Since fuel cells can generate electricity over extended periods, they exhibit strong potential for wearable applications in terms of sustainability. Wearable biofuel cells typically utilize enzymes or electroactive bacteria as catalysts [3]. Microbial fuel cells (MFCs) convert chemical energy into electrical energy by consuming organic matter. Because they can utilize organic compounds present in wastewater, they are considered environmentally friendly and promising for environmental remediation [4]. MFCs are also gaining attention as point-of-care diagnostic devices, particularly in resource-limited environments, since organic substrates required for microbial electricity generation are widely available. However, the operational lifetime of MFCs is still insufficient for practical electronic applications. Therefore, photosynthetic microbial fuel cells, which can operate for longer durations, have attracted increasing interest [5]. Photosynthetic microbial solar cells use oxygenic photosynthetic organisms as biocatalysts to convert abundant and renewable light energy into electrical power. Although the power output is lower in dark conditions compared to illuminated environments, electricity can still be generated through the respiratory activity of photosynthetic microorganisms. In addition, these systems possess self-sustainable characteristics due to their ability to undergo continuous biological repair [6].

Conventional MFCs rely on expensive proton exchange membranes, noble metal catalysts such as platinum for the cathode, and mediators to enhance electron transfer in the catholyte. These requirements increase installation and operational costs while limiting power output, hindering commercialization [7]. Proton exchange membranes also complicate fabrication processes and increase overall costs. As an alternative, co-laminar flow-based fuel cells utilize a liquid–liquid interface formed between laminar electrolyte streams in microchannels, eliminating the need for membranes [8]. Such membraneless microfluidic fuel cells enable easy miniaturization and simplified fabrication, resulting in low-cost production. The use of chemical oxidants such as potassium ferricyanide as electron acceptors in the catholyte improves cathodic overpotential and reduction reactions [9]; however, these chemicals are expensive and environmentally unfriendly. Alternatively, air-cathodes that utilize oxygen from air without mediators are low-cost, environmentally friendly, and sustainable, but they suffer from high overpotential and limited performance. Although not definitively superior to air-cathodes, microalgae have been explored as alternative biocatalysts to replace platinum at the cathode [7]. In microalgae biocathodes, oxygen is generated in the aqueous medium, which reduces cathodic overpotential at the electrode surface [10].

The electrogenic capacity of photosynthetic bacteria is generally much lower than that of electroactive bacteria used in MFCs [11]. In particular, photosynthetic MFCs suffer from relatively low power density due to high internal resistance, primarily caused by insufficient electron transfer between the biofilm and the anode [9]. However, co-cultured microbial solar cells combine the advantages of high power density from MFCs and long operational lifetime from photosynthetic microorganisms [12]. These systems employ symbiotic cultures of heterotrophic bacteria and photosynthetic microorganisms. The heterotrophic bacteria produce carbon dioxide through respiration, which is subsequently utilized by the photosynthetic microorganisms during photosynthesis [13]. Miniaturization of devices enhances performance by increasing the surface-area-to-volume ratio and mass transfer rates [14,15]. Although miniaturized photosynthetic MFCs have been developed, they still suffer from limited lifetimes and relatively low power output and voltage [2]. Most reported miniature photosynthetic MFCs are dual-chamber systems with membranes, resulting in low performance relative to their high fabrication cost [9,16]. On the other hand, single-chamber membraneless miniature systems typically operate in batch mode, where the supply of CO_2_ and N_2_ required for biofilm growth is limited [9,17], leading to insufficient operational lifetime [18]. Furthermore, most miniaturized photosynthetic MFCs lack flexibility, making them unsuitable for wearable applications. Flexible MFCs based on paper substrates have been reported, benefiting from high microbial surface area due to their porous structure [19]. However, paper substrates suffer from poor durability when exposed to liquid electrolytes, limiting long-term operation. In contrast, textiles offer excellent elasticity and mechanical resilience, making them more suitable as substrates for wearable fuel cells [20]. In addition, textile-based systems enable the development of self-driven, continuous-mode co-laminar flow fuel cells without the need for membranes or external pumps. Therefore, textile-based fuel cells are expected to contribute to cost reduction through device miniaturization and simplified fabrication processes.

In this study, to the best of our knowledge, a photosynthetic microbial fuel cell utilizing a microalgae photoreactor as a biocathode was developed for the first time as an alternative to platinum-based systems. To overcome the limitations of miniaturized photosynthetic MFCs, the device was designed with a continuous-flow membraneless co-laminar configuration. A textile substrate with superior durability compared to paper was employed to achieve flexibility. Channels and electrodes were fabricated in a single layer on the textile substrate using a screen-printing technique. Performance evaluations were conducted for different cathodic reduction strategies to verify the enhancement effect of the microalgae biocathode. A multilayered co-cultured microbial system was used as the anodic catalyst. Additional performance tests were carried out for various anodic microbial configurations to determine the optimal photosynthetic microbial fuel cell design. Furthermore, long-term operation, bending, and tensile tests were performed to evaluate durability and mechanical flexibility. The novelty of this work lies in the integration of a textile-based flexible substrate, a membraneless co-laminar microfluidic architecture, a multilayered co-cultured bioanode, and a microalgae biocathode into a single platform, enabling continuous-flow, self-sustaining operation that has not been previously demonstrated. Recent advances in microfluidic bio-photovoltaics have achieved varying degrees of success; for instance, systems utilizing optical interactions reached 41.4 μW cm^−2^ [21], and 3D bio-anode configurations reported 5.34 μW cm^−2^ [22]. Although macro-scale community-based cells [23] achieve higher outputs (83.6 μW cm^−2^), they lack the flexibility and micro-scale integration required for wearables. Our work distinguishes itself by integrating a textile-based, membraneless co-laminar architecture with a dual-biocatalyst system (co-cultured anode and microalgae cathode). This synergy allows us to overcome the common trade-off between flexibility and power density, achieving 17 μW cm^−2^—a competitive value for a flexible, self-driven system.

## 2. Materials and Methods

### 2.1. Design and Fabrication of Biosolar Cell

**Figure 1:**
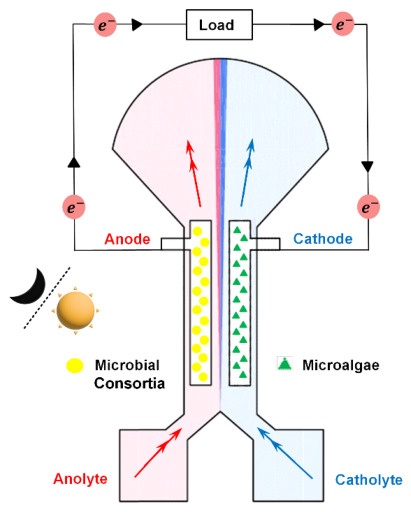
Schematic illustration of the textile-based membraneless microfluidic microalgae–microbial solar cell (μmMSC) with a co-laminar flow configuration. The device consists of a microbial co-culture bioanode and a microalgae biocathode integrated within a single-layer textile substrate.

**Figure A1:**
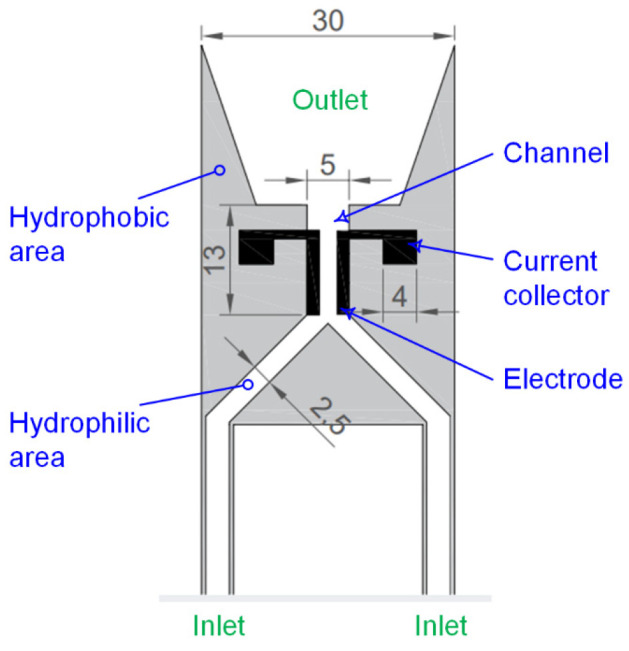
Design layout of the textile-based microfluidic microalgae–microbial solar cell (μmMSC). All dimensions are given in millimeters.

Based on previous studies on textile-based biofuel cells, a microfluidic microalgae–microbial solar cell (μmMSC) was designed [24]. Figure 1 shows a schematic diagram of the single-layer μmMSC. It features a Y-shaped channel configuration, and the dimensions of the cell were set to be identical to those in the previous study. The device consists of two inlets and one outlet. The electrodes embedded within the channel have a width of 1.5 mm, a length of 10 mm, and an inter-electrode spacing of 2 mm. Further details of the cell design are provided in Figure A1. On the hydrophilic single-layer substrate, all regions except for the channel, inlets, and outlet were modified to be hydrophobic, ensuring that the electrolyte flows only through the channel. As a flexible textile substrate, 30s cotton (100% cotton, WD1025, Zentex Co., Ltd., Daegu, Republic of Korea) was used. For hydrophobic treatment, the substrate was impregnated with Ecoflex (Ecoflex 00-30, Smooth-On Inc., Macungie, PA, USA), a hydrophobic material. Ecoflex was first patterned onto the textile substrate via silk-screen printing using a squeegee to ensure selective hydrophobicity. The Ecoflex-impregnated textile substrate was subsequently dried in an oven at 80 °C for 30 min.

As the electrode material formed on the substrate, PEDOT:PSS paste (Clevios™ S V4 STAB, Heraeus, Germany) was used. PEDOT:PSS is a conductive polymer material with excellent electrical conductivity, high optical transparency, and superior flexibility. When Ag_2_O was used as a solid oxidant at the cathode, 1 mL of PEDOT:PSS paste was mixed with 0.05 g of Ag_2_O and subjected to a grinding process for 15 min to obtain a paste form. Using an OHP film mask patterned by a laser cutting machine (Speedy 100, Trotec, Heinsberg, Germany), the prepared pastes were screen-printed onto the substrate in the shape of the electrodes. To enhance the electrical conductivity of the electrodes, a CNTs paste composed of a mixture of multi-walled carbon nanotubes and single-walled carbon nanotubes was additionally screen-printed on top of the initially coated electrodes. The CNTs paste was prepared and screen-printed following the same procedure as described in the previous study [25].

### 2.2. Preparation of Catalyst and Electrolytes

Since the cell is configured as a single layer, the microbial catalysts are exposed to the atmosphere. Therefore, aerobic microorganisms were utilized. The preparation of Luria–Bertani (LB) broth media and minimal media required for microbial cultivation, as well as the cultivation of *Shewanella oneidensis* MR-1 (BAA-1096, ATCC^®^, Manassas, VA, USA), were conducted following the same procedures as in a previous study [26]. The microorganisms were cultured in sterilized L-Broth media at 25 °C for 48 h. The L-Broth media was prepared by mixing 25 g of LB Miller (BD Difco, Franklin Lakes, NJ, USA) with 1 L of deionized (DI) water. *Bacillus subtilis* (KCTC-3038, Jeongeup-si, Republic of Korea) was cultured using the same method as *S. oneidensis*. The microalga *Scenedesmus obliquus* (KCTC-AG20491, KCTC, Jeongup-si, Republic of Korea) was cultured in BG-11 media, which was prepared by dissolving 1.64 g of BG-11 Broth (MB-B0827, Ducksan General Science, Seoul, Republic of Korea) in 1 L of DI water. The photosynthetic microorganism (cyanobacteria) *Synechocystis* sp. PCC 6803 (ATCC, Manassas, VA, USA) was cultured in the same manner as the microalga (μALG) *S. obliquus*. Both *Synechocystis* sp. and *S. obliquus*, which perform photosynthesis, were incubated in a chamber with alternating light and dark conditions at 12-h intervals to promote growth. All microorganisms were cultured in 15 mL of medium, after which the microbial pellets and media were separated by centrifugation. The *S. oneidensis* MR-1 (MR1) pellet was resuspended in 5 mL of fresh minimal media, while the pellets of *S. obliquus* and *Synechocystis* sp. (*S*. sp.) were resuspended in 1 mL of BG-11 media. *B. subtilis* (*B. sub*) was resuspended in 1 mL of L-Broth media. The microbial concentration in the mixtures was measured using a double-beam spectrophotometer (UV-1800, Shimadzu, Japan). The absorbance values measured at a wavelength of 600 nm (OD_600_) were 1.5 (±0.03) for *S. oneidensis* (5 mL), 1.403 (±0.0015) for *B. subtilis* (1 mL), 1.2 (±0.036) for *S. obliquus* (1 mL), and 0.971 (±0.0125) for *Synechocystis* sp. (1 mL). Finally, 5 μL of the pellet-containing media was deposited onto the electrode using a micropipette.

To prepare Luria broth (LB)-based agar used for forming the microbial co-cultured biocatalyst, 100 mL of L-Broth media was mixed with 1.5 g of agar powder (Daejung Chemical & Metals, Siheung-si, Republic of Korea) and sterilized using an autoclave. The prepared LB-based agar solution was then cooled to approximately 35 °C and poured into a mold before solidification. The mold was fabricated using a 1 mm-thick acrylic plate with openings matching the electrode surface dimensions (1.5 mm × 10 mm). Excess solution overflowing the mold was removed, and the agar was allowed to solidify by cooling at 25 °C for 10 min, resulting in solid-state agar layers. The prepared LB-based agar layers were then separated from the mold, sealed with paraffin tape (PARAFILM M, Bemis Co., Inc., Neenah, WI, USA), and stored under refrigeration until use. *S. oneidensis* was first deposited onto the anode, followed by placement of an agar layer on top. Subsequently, *B. subtilis* was deposited onto the agar layer, and another agar layer was placed above it. Finally, *Synechocystis* sp. was applied to the top agar layer, completing the microbial co-culture configuration.

In experiments where either a microbial co-culture or only cyanobacteria were used as the anodic biocatalyst, BG-11 media was employed as the anolyte. When *S. oneidensis* alone was used as the anodic biocatalyst, minimal media was utilized. As the catholyte, a 0.1 M phosphate-buffered saline (PBS) solution (P4417-100TAB, Sigma-Aldrich, Burlington, MA, USA) with a pH of 7.4 was used in all experiments. The morphology of the electrode surface and the inoculated biocatalysts was observed using a field emission scanning electron microscope (FE-SEM; MIRA3, TESCAN, Brno, Czech Republic). For FE-SEM observation, the microorganism-inoculated electrodes were fixed by immersing them in a 2% glutaraldehyde solution for 24 h. Subsequently, the samples were dehydrated by immersing them in 50% and 100% ethanol for 5 min each.

### 2.3. Experimental Set-Up

**Figure A2:**
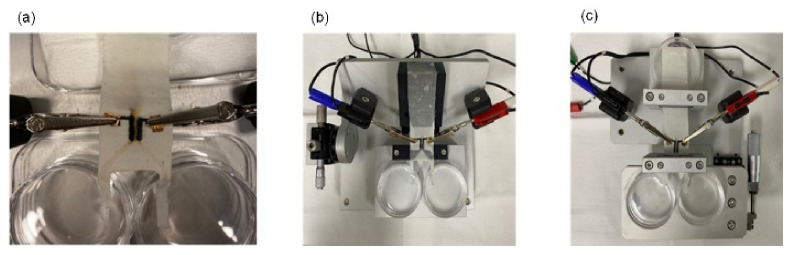
Experimental setup: (**a**) electrochemical performance measurement; (**b**) bending test configuration; and (**c**) tensile test configuration.

A chamber capable of simulating day and night conditions required for the performance evaluation of the μmMSC was fabricated in-house. LED lights (PHI WEB 60W A60 E26 922-65 RGB, PHILIPS Korea, Seoul, Republic of Korea) mounted on the ceiling were turned on and off at 12-h intervals. Using an illuminometer (TES1339 Light Meter Pro, TES Electrical Electronic Corp., Rui Guang, Taiwan), the illumination was adjusted to 3000 lux at a height of 30 mm from the base where the μmMSC was placed. The μmMSC was connected to a potentiostat (Ivium-n-stat, Ivium Technologies Inc., Eindhoven, The Netherlands) for performance testing. A 5 × 5 mm copper tape was attached to the current collector of the μmMSC and brought into contact with the connection wire clip of the potentiostat. To prevent the flexible μmMSC from bending under the weight of the clip, the wiring was fixed using a custom-fabricated jig (Figure A2a). Subsequently, the inlets of the μmMSC were immersed separately into Petri dishes containing the anolyte and catholyte, and the experiments were carried out.

Polarization curves were obtained using a galvanostatic linear sweep method (at a sweep rate of 0.5 μA s^−1^), and the power density was calculated accordingly. An Ag/AgCl reference electrode was inserted into the electrolyte discharged from the outlet of the μmMSC, and the working and counter electrodes of the potentiostat were connected to the cathode and anode, respectively, to measure the cathode potential (V_(cathode)_). The anode potential (V_(anode)_) was calculated using the relation V_(anode)_ = V_(cathode)_ − V_(cell)_ [27]. Chronoamperometry measurements were conducted by applying 0.1 V. To evaluate the flexibility of the μmMSC, bending and tensile tests were performed using an angle stage (SR1-417-L1, Sciencetown, Incheon, Republic of Korea) and a linear stage (SS1-48L, Sciencetown, Incheon, Republic of Korea), respectively (Figure A2b,c). All experimental measurements were conducted at least in triplicate to ensure reproducibility. Data are expressed as the mean ± standard deviation (SD), and error bars in the plots represent the SD of the measurements. The chosen operating conditions were determined based on preliminary optimization and established literature. The light intensity of 3000 lux was selected to support robust photosynthesis of the microalgae and cyanobacteria while avoiding photo-oxidative stress. The application of 0.1 V during chronoamperometry was intended to promote steady-state biofilm development by providing a stable electrochemical gradient. Furthermore, the continuous-mode operation was employed to ensure a constant supply of substrates (CO_2_ and nutrients) to the co-cultured biofilm, overcoming the mass-transfer limitations typically observed in batch-mode microfluidic systems.

## 3. Results and Discussion

### 3.1. Performance Depending on Anodic Biocatalyst

In this study, the anodic catalysts were evaluated under conditions without externally supplied electron donors, in order to reflect the intended operation of a self-sustaining bio-solar cell driven solely by naturally available energy sources such as light. To evaluate the performance of the anode, experiments were conducted using different anodic catalyst configurations. On the PEDOT:PSS/CNTs anode electrode—where carbon nanotubes were coated onto PEDOT:PSS—either photosynthetic bacteria (*Synechocystis* sp.) or a co-culture of heterotrophic and photosynthetic bacteria was used as the biocatalyst. BG-11 media was used as the anolyte in these cases. As an alternative biocatalyst, the electroactive bacterium *Shewanella oneidensis* MR-1 was employed, with minimal media used as the anolyte. Since the objective of the developed fuel cell was to achieve a self-sustainable system, the minimal media did not contain an electron donor (lactate). For all anode types, a 0.1 M phosphate-buffered saline (PBS) solution (pH 7.4) was consistently used as the catholyte. In addition, the same cathode was used in all cases, consisting of carbon nanotubes (CNTs) coated onto PEDOT:PSS mixed with Ag_2_O.

**Figure A3:**
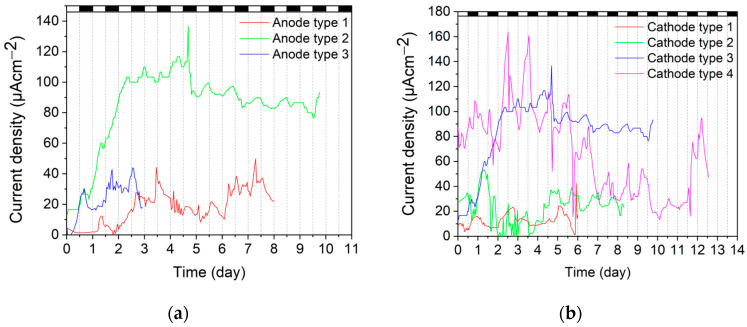
Chronoamperometry responses for different configurations: (**a**) anodic biocatalysts (type 1: *Synechocystis* sp.; type 2: MR1–*B. subtilis*–*Synechocystis* sp.; type 3: MR1); (**b**) cathode materials (type 1: PEDOT:PSS/CNTs; type 2: PEDOT:PSS/CNTs/μALG; type 3: PEDOT:PSS:Ag_2_O/CNTs; type 4: PEDOT:PSS:Ag_2_O/CNTs/μALG).

The results of chronoamperometry performed for each anode type are shown in Figure A3a, where the co-cultured biocatalyst (MR1-*B.sub*-*S*.sp.) clearly exhibits a significantly higher current density. For the photosynthetic microorganisms, after approximately 3 days—when the current density reached a stable trend—the average values of the maximum and minimum current densities were calculated over 24-h intervals. In the case of the fuel cell using electroactive bacteria, since its operation lasted only about 3 days, the average of the maximum and minimum current densities was calculated for each 24-h period up to day 3 (Table A1). For both the photosynthetic bacteria (*S*. sp.) and the microbial co-culture (MR1-*B.sub*-*S*.sp.), higher current densities were observed under illuminated conditions compared to dark conditions. In contrast, the electroactive bacteria (MR1), which did not receive an external electron donor, exhibited relatively low current density and a shorter operational duration due to the lack of electron donor supply.

**Figure 2:**
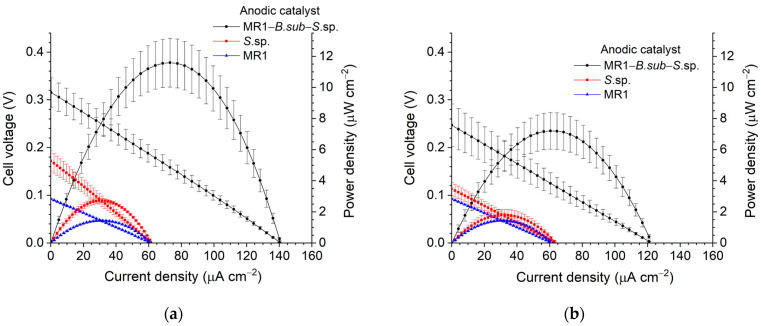
Polarization and power density curves for different anodic biocatalysts: (**a**) under illumination and (**b**) under dark conditions.

After conducting chronoamperometry for 3 days, polarization curves were measured (Figure 2). The light and dark conditions were defined as 9–11 h after the light was turned on and 11–12 h after it was turned off, respectively. Compared to the light condition, the maximum power density under dark conditions decreased by approximately 37% and 33% for the co-cultured biocatalyst and the photosynthetic bacteria catalyst, respectively. In contrast, the electroactive bacteria catalyst exhibited nearly identical performance under both light and dark conditions. Regardless of illumination conditions, the co-cultured biocatalyst demonstrated the best performance under self-sustaining conditions among all biocatalysts in terms of peak power density, maximum current density, and open circuit voltage (OCV). While the photosynthetic bacteria catalyst showed performance similar to that of the electroactive bacteria catalyst under dark conditions, it exhibited higher power density and OCV under illuminated conditions. This improvement is attributed to the role of riboflavin produced by *Bacillus subtilis* (*B. sub*) in the co-culture, which acts as an electron shuttle. Electrons generated by cyanobacteria through photosynthesis are transferred to the *Shewanella* layer, while metabolic byproducts produced by cyanobacteria are delivered through the LB agar to the heterotrophic microbial layer, where they are utilized as nutrients. This synergistic interaction is considered to enhance the overall power density [28].

**Figure A4:**
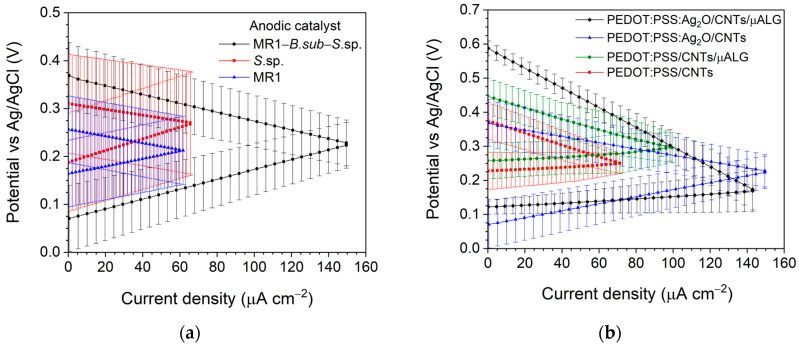
Half-cell electrode performance under illumination for (**a**) different anodic biocatalysts and (**b**) different cathode materials.

Figure A4 shows the individual electrode potentials measured using a reference electrode under light illumination. The half-cell electrode performance for various anodic biocatalysts was evaluated under identical cathode and catholyte conditions, and therefore reflects the influence of the anodic catalyst and anolyte. Based on the half-cell potential results (Figure A4a), the co-cultured biocatalyst exhibits the lowest anodic potential across the measured current density range. This lower potential indicates a significantly reduced anodic overpotential, facilitating more efficient substrate oxidation. This trend aligns with the full-cell polarization data, confirming that the synergistic effect of the co-culture enhances the overall electrochemical flux. It should be noted that the anodic catalysts were not tested under fully identical conditions, and thus the observed performance differences cannot be solely attributed to intrinsic catalytic activity.

### 3.2. Performance Depending on Cathode Type

To determine the optimal configuration of the photosynthetic microbial fuel cell, performance evaluation experiments were conducted using various types of cathode catalysts. The anode employed the co-cultured biocatalyst, which had demonstrated the best performance. As the air cathode, only PEDOT:PSS/CNTs was used. In another cathode configuration, Ag_2_O oxidant was incorporated into the PEDOT:PSS of the air cathode (PEDOT:PSS:Ag_2_O/CNTs), while in a different case, a microalgae (μALG) catalyst was inoculated onto PEDOT:PSS/CNTs (PEDOT:PSS/CNTs/μALG). In addition, a cathode combining both approaches was fabricated, where microalgae were inoculated onto PEDOT:PSS:Ag_2_O/CNTs (PEDOT:PSS:Ag_2_O/CNTs/μALG). The anolyte was consistently BG-11 media for all cases, and the catholyte was uniformly a 0.1 M PBS solution (pH 7.4).

According to the results of the chronoamperometry measurements, the use of the Ag_2_O oxidant resulted in a significantly higher current density (Figure A3b). Since cyanobacteria were used as the anodic catalyst, all cathode configurations exhibited differences in current density between light and dark conditions. After approximately 3 days, when the current density reached a stable trend, the average values of the maximum and minimum current densities measured over each 24-h period were calculated (Table A2). From the averaged maximum current density values, it can be inferred that the use of microalgae enhances current density under illuminated conditions. On the other hand, the incorporation of Ag_2_O improved the power density under both light and dark conditions.

**Figure 3:**
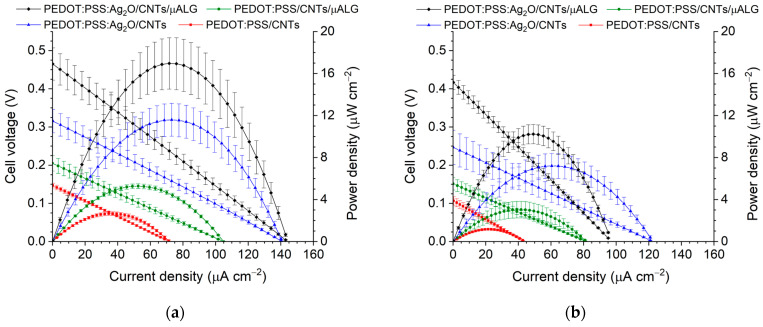
Polarization and power density curves for different cathode configurations: (**a**) under illumination and (**b**) under dark conditions.

According to the polarization curves for each cathode configuration (Figure 3), the maximum power density under dark conditions decreased compared to illuminated conditions by approximately 57% for PEDOT:PSS/CNTs, 42% for PEDOT:PSS/CNTs/μALG, 38% for PEDOT:PSS:Ag_2_O/CNTs, and 40% for PEDOT:PSS:Ag_2_O/CNTs/μALG. These results suggest that the Ag_2_O oxidant contributes to maintaining the maximum power density performance under dark conditions. The Ag_2_O solid oxidant provides a two-phase cathodic reaction involving Ag_2_O and water [29]. Therefore, limitations such as low reactant collision probability or low aqueous oxygen solubility can be mitigated. As a result, cathodic overpotential is reduced, leading to improved cell performance. Similar to the chronoamperometry results, the use of microalgae enhances the maximum current density under illuminated conditions. This is attributed to the oxygen generated through photosynthesis by the microalgae, which promotes the reduction reaction at the cathode electrode [7]. Ultimately, the cathode utilizing both Ag_2_O and microalgae exhibited the best performance, showing a maximum current density of 144 ± 17.0 μA cm^−2^ and a peak power density of 17 ± 2.5 μW cm^−2^ under illuminated conditions.

Figure A4b presents the cathode potentials measured for different cathodic materials under identical anodic and electrolyte conditions, enabling a direct comparison of their electrochemical performance. Among the tested materials, the electrode modified with Ag_2_O and microalgae (μALG) maintains the highest cathode potential at a given current density. A higher cathode potential in this configuration signifies a minimized cathodic overpotential and superior reduction kinetics. This improved performance can be attributed to the synergistic effect of Ag_2_O and microalgae. Ag_2_O is known to facilitate efficient electron transfer and catalytic oxygen reduction, while the microalgae contribute to enhanced light-driven charge generation and provide bio-compatible active sites, thereby promoting the overall cathodic reaction under illumination. Consistent with the half-cell measurements, the polarization curve results also demonstrate that the Ag_2_O- and μALG-modified electrode achieves the highest power density and current density. These findings confirm that the incorporation of Ag_2_O and microalgae significantly enhances the cathodic performance and contributes to the improved overall performance of the bio-photoelectrochemical system.

The enhanced performance of the proposed μmMSC can be attributed to the synergistic electrochemical and metabolic interactions at both the anode and cathode. At the anode, the co-cultured system combines direct electron transfer by *Shewanella oneidensis MR-1* with mediated electron transfer facilitated by redox-active metabolites (e.g., riboflavin) produced by *Bacillus subtilis*, while *Synechocystis* sp. supplies photosynthetically derived organic compounds. In addition, the spatially layered configuration enables efficient recycling of metabolic byproducts, where CO_2_ generated by heterotrophic bacteria is utilized by photosynthetic microorganisms, and oxygen produced via photosynthesis supports aerobic metabolism. This internal cycling contributes to reduced anodic overpotential and sustained current generation. At the cathode, microalgae function as an in situ oxygen source under illumination, enhancing oxygen reduction reaction kinetics, whereas Ag_2_O acts as a solid-state oxidant that facilitates charge transfer and maintains cathodic performance even under dark conditions. The combination of these mechanisms results in reduced cathodic limitations and improved overall cell performance compared to single-component systems.

### 3.3. Electrode Surface Morphology and Sustainability

**Figure 4:**
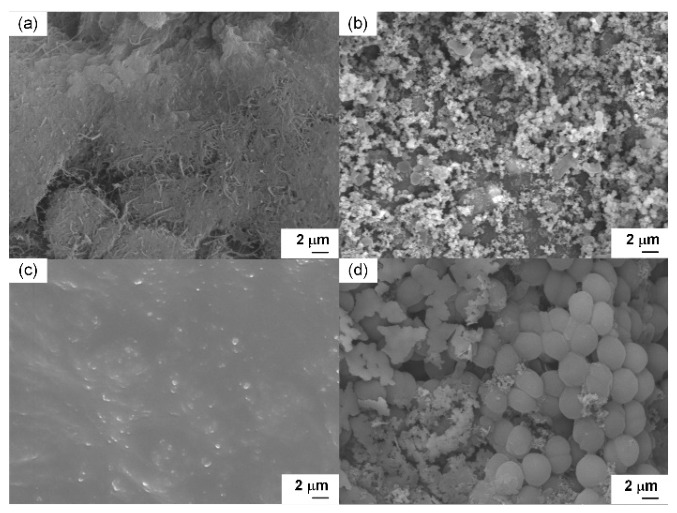
FE-SEM images of electrode surfaces: (**a**) PEDOT:PSS:Ag_2_O/CNTs-coated cathode without microalgae; (**b**) microalgae (*S. obliquus*) inoculated on PEDOT:PSS:Ag_2_O/CNTs cathode; (**c**) PEDOT:PSS/CNTs/MR1/Agar/*B.sub*/Agar anode without *Synechocystis* sp.; and (**d**) *Synechocystis* sp. inoculated on the multilayered PEDOT:PSS/CNTs/MR1/Agar/*B.sub*/Agar anode after operation.

**Figure 5:**
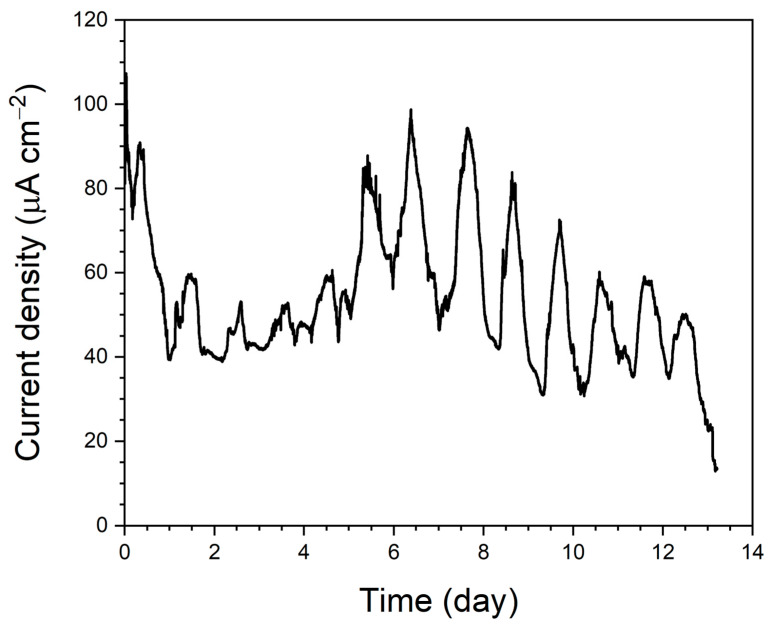
Chronoamperometry response of the μmMSC under continuous electrolyte flow conditions.

A constant voltage of 0.1 V was applied to the fabricated μmMSC for 5 days to induce biofilm formation on the electrode surfaces, after which the electrodes were observed using FE-SEM. Figure 4a shows an FE-SEM image of the cathode, where PEDOT:PSS:Ag_2_O/CNTs was coated onto a textile substrate without microbial inoculation. Figure 4b presents the FE-SEM image after microalgae were inoculated onto the coated cathode and a biofilm was formed. Figure 4c shows the anode structure (PEDOT:PSS/CNTs/MR1/Agar/*B.sub*/Agar) without inoculation of *Synechocystis* sp. (*S*. sp.). Figure 4d shows the same anode after inoculation with *Synechocystis* sp. and operation of the μmMSC to allow biofilm formation. By comparing the images in Figure 4, it can be confirmed that both the microalgae and photosynthetic microorganisms were successfully inoculated. Figure 4a reveals the morphology of the PEDOT:PSS:Ag_2_O/CNTs-coated textile, showing that the conductive materials successfully wrapped around the individual cotton fibers, creating a hierarchical porous structure. This high surface-area-to-volume ratio is advantageous for microbial attachment. In Figure 4b, the microalgae (*S. obliquus*) are observed to be firmly embedded within the CNT/polymer matrix. Similarly, Figure 4d displays the anode surface after 5 days of operation. The *Synechocystis* sp. cells are integrated with the underlying multilayered microbial structure. The close physical proximity between the microbial cell membranes and the CNT network, as seen in these images, is critical for facilitating both direct and mediated electron transfer. Finally, a chronoamperometry test was conducted on the μmMSC composed of the best-performing configuration: PEDOT:PSS/CNTs/MR1/Agar/*B.sub*/Agar/*S*.sp. anode and PEDOT:PSS:Ag_2_O/CNTs/μALG cathode (Figure 5). The experimental results demonstrated that the developed μmMSC operated continuously for approximately 13 days.

Compared to macroscale co-culture systems, such as the spatially engineered microbial consortium [28], which achieved approximately 60 μW cm^−2^ and 120 μA cm^−2^ with an operational duration of 18 days, the present system shows lower power density (17 μW cm^−2^), higher current density (144 μA cm^−2^), and slightly shorter operational stability (approximately 13 days). However, it should be noted that the macroscale system benefits from larger electrode surface areas and reduced internal resistance, which are difficult to achieve in microfluidic configurations. Most high-performance systems rely on rigid architectures and complex membrane-separated chambers. In contrast, our μmMSC utilizes a single-layer textile substrate, enabling sustainable power generation in a flexible form factor suitable for wearable electronics. Despite its flexibility, the μmMSC exhibits a limited power density compared to macro-scale devices, primarily due to the higher internal resistance inherent in the porous textile substrate. The effective surface area of the screen-printed electrodes may not fully capture the total electrochemical flux generated by the multilayered microbial consortium, thus limiting the overall charge transfer efficiency.

### 3.4. Flexibility Assay

**Figure 6:**
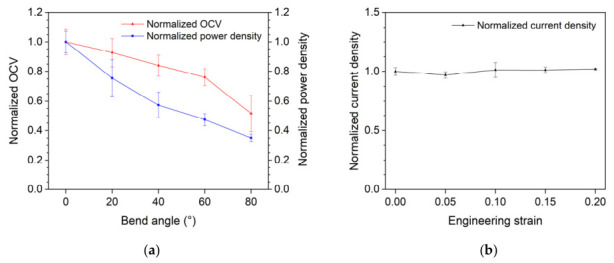
Mechanical flexibility performance of the μmMSC: (**a**) normalized open-circuit voltage (OCV) and power density as a function of bending angle; (**b**) normalized current density as a function of applied tensile engineering strain.

**Figure A5:**
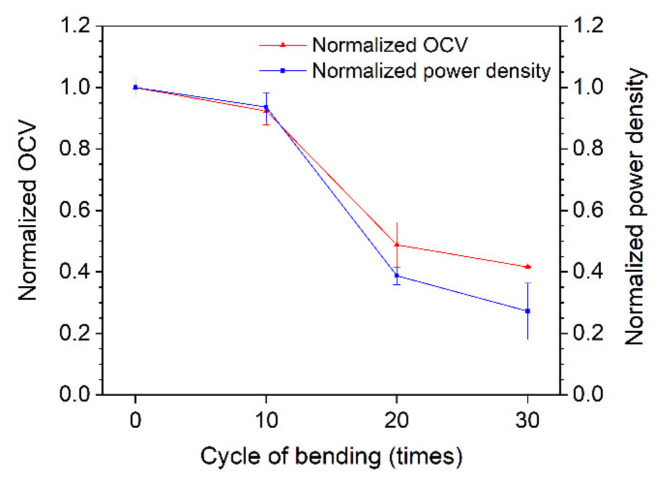
Normalized open-circuit voltage (OCV) and power density as a function of the number of bending cycles (bending angle: 20°).

A flexibility assay was conducted on the μmMSC composed of a PEDOT:PSS/CNTs/ MR1/Agar/*B.sub*/Agar/*S*.sp anode and a PEDOT:PSS:Ag_2_O/CNTs/μALG cathode. The assay was performed through tensile and bending tests, and performance evaluation was carried out under continuous flow conditions in an illuminated environment. Whenever the test conditions were changed, chronoamperometry was conducted for 15 min while applying a voltage to ensure stabilization of the output current, after which polarization curves were measured. For the bending test, the μmMSC was bent at angles of 0°, 20°, 40°, 60°, and 80°, and the open circuit voltage (OCV) and maximum power density were measured. The results, normalized to the performance at 0° (no bending), are shown in Figure 6a. As the bending angle increased, the cell performance decreased in an almost linear manner. The reduction in power density was more pronounced than that in OCV. At a bending angle of 80°, the power density decreased to approximately 65% of its original value. A bending fatigue test was also performed by repeatedly bending the device from 0° to 20° for 10 cycles at a time. After every 10 cycles, polarization curves were obtained. The results of three repeated measurements are shown in Figure A5. After 10 bending cycles, both power density and OCV decreased by less than 5%. However, after 20 cycles, a significant decrease in performance was observed (power density reduced by approximately 60%), and further degradation occurred with repeated bending up to 30 cycles. This is likely due to deformation of the agar layer caused by repeated bending of the device. These results indicate that while the device exhibits initial flexibility, its mechanical durability under repeated deformation is currently limited, particularly under bending fatigue conditions.

The cell was clamped to an initial length of 40 mm, and a stretching test was performed by applying tensile strain along the channel length direction using an X-stage (Figure 6b). The maximum power density was measured at strain levels of 0%, 5%, 10%, 15%, and 20%. The variation in current density was minimal up to a strain of 20%. As the cell was stretched, a reduction in the distance between the electrodes was observed. It is considered that the performance enhancement caused by this effect and the performance degradation due to electrode deformation offset each other. Therefore, tensile deformation is not regarded as having a significant impact on cell performance. For effective operation of the co-cultured microbial biocatalyst, heterotrophic and photosynthetic bacteria must be spatially separated [13]. For this purpose, LB agar was placed between the bacterial layers. However, the agar used in this configuration has the lowest flexibility among the components of the μmMSC and thus is considered to significantly contribute to performance degradation under flexible deformation. If a method can be developed to prevent direct contact between bacteria using a material more flexible than agar, the proposed μmMSC would become more suitable for wearable device applications.

## 4. Conclusions

In this study, a flexible and durable bio-solar cell was developed as a power source for miniaturized wearable devices. The use of a textile substrate provided excellent mechanical flexibility, while the microfluidic configuration enabled device miniaturization. However, further improvements in mechanical durability are required for practical wearable applications, particularly by replacing the agar layer with more flexible and durable materials. Although the power density remains lower than that of conventional electroactive microbial fuel cells, the proposed system demonstrated a key advantage in long-term operation, sustaining performance for over 13 days through the use of photosynthetic microorganisms. Furthermore, the agar-separated co-culture system enabled continuous power generation without the need for external electron donors. The incorporation of a microalgae biocathode further enhanced overall performance. However, the current output is still insufficient for direct application as a standalone power source. Future work should focus on stacked configurations and optimized designs to improve power output while maintaining compactness and flexibility. Future improvements will focus on electrical scaling through series and parallel configurations and design changes. Specifically, implementing diverging channels and multiple inlets will be explored to expand diffusion areas and suppress depletion layers, thereby enhancing mass transport and overall power density.

## Data Availability

The original contributions presented in this study are included in the article. Further inquiries can be directed to the corresponding author.

## Appendix A

**Table A1:** Chronoamperometric performance of the μmMSC with different anodic biocatalyst configurations. The highest and lowest current densities were calculated from stabilized 24 h cycles.

Anodic Configuration	Highest Current Density (μA cm^−2^)	Lowest Current Density (μA cm^−2^)
PEDOT:PSS/CNTs/ *S*.sp.	32.2 ± 8.81	16.1 ± 6.62
PEDOT:PSS/CNTs/ MR1-*B.sub*-*S*.sp.	103.3 ± 15.4	89.8 ± 8.61
PEDOT:PSS/CNTs/ MR1	36.3 ± 5.41	11.7 ± 8.21

**Table A2:** Chronoamperometric performance of the μmMSC with different cathode configurations. The highest and lowest current densities were obtained under stabilized operating conditions.

Cathodic Configuration	Highest Current Density (μA cm^−2^)	Lowest Current Density (μA cm^−2^)
PEDOT:PSS/CNTs	17.6 ± 4.68	7.7 ± 2.16
PEDOT:PSS/CNTs/μALG	32.7 ± 6.90	16.7 ± 7.69
PEDOT:PSS:Ag_2_O/CNTs	103.3 ± 15.41	89.8 ± 8.61
PEDOT:PSS:Ag_2_O/CNTs/μALG	119.6 ± 26.16	67.1 ± 12.37

## References

[1] Huang X., Zhang L., Zhang Z., Guo S., Shang H., Li Y., Liu J. (2019). Wearable biofuel cells based on the classification of enzyme for high power outputs and lifetimes. Biosens. Bioelectron..

[2] Mohammadifar M., Tahernia M., Choi S. (2020). A miniaturized, self-sustaining, and integrable bio-solar system. Nano Energy.

[3] Chen G., Li Y., Bick M., Chen J. (2020). Smart textiles for electricity generation. Chem. Rev..

[4] Trapero J.R., Horcajada L., Linares J.J., Lobato J. (2017). Is microbial fuel cell technology ready? An economic answer towards industrial commercialization. Appl. Energy.

[5] Stephens S., Mahadevan R., Allen D.G. (2021). Engineering photosynthetic bioprocesses for sustainable chemical production: A review. Front. Bioeng. Biotechnol..

[6] Bombelli P., Müller T., Herling T.W., Howe C.J., Knowles T.P. (2015). A high power-density, mediator-free, microfluidic biophotovoltaic device for cyanobacterial cells. Adv. Energy Mater..

[7] Lee D.-J., Chang J.-S., Lai J.-Y. (2015). Microalgae-microbial fuel cell: A mini review. Bioresour. Technol..

[8] Wu B., Xu X., Dong G., Zhang M., Luo S., Leung D.Y.C., Wang Y. (2024). Computational modeling studies on microfluidic fuel cell: A prospective review. Renew. Sust. Energ. Rev..

[9] Lee H., Choi S. (2015). A micro-sized bio-solar cell for self-sustaining power generation. Lab Chip.

[10] Fischer F. (2018). Photoelectrode, photovoltaic and photosynthetic microbial fuel cells. Renew. Sust. Energ. Rev..

[11] McCormick A.J., Bombelli P., Bradley R.W., Throne R., Wenzel T., Howe C.J. (2015). Biophotovoltaics: Oxygenic photosynthetic organisms in the world of bioelectrochemical systems. Energy Environ. Sci..

[12] Liu L., Choi S. (2017). Self-sustaining, solar-driven bioelectricity generation in micro-sized microbial fuel cell using co-culture of heterotrophic and photosynthetic bacteria. J. Power Sources.

[13] He Z., Kan J., Mansfeld F., Angenent L.T., Nealson K.H. (2009). Self-sustained phototrophic microbial fuel cells based on the synergistic cooperation between photosynthetic microorganisms and heterotrophic bacteria. Environ. Sci. Technol..

[14] Liu L., Choi S. (2021). Miniature microbial solar cells to power wireless sensor networks. Biosens. Bioelectron..

[15] Cho C.-H., Cho W., Ahn Y., Hwang S.-Y. (2007). PDMS-glass serpentine microchannel chip for time domain PCR with bubble suppression in sample injection. J. Micromech. Microeng..

[16] Furukawa Y., Moriuchi T., Morishima K. (2006). Design principle and prototyping of a direct photosynthetic/metabolic biofuel cell (DPMFC). J. Micromech. Microeng..

[17] Ha J.-G., Song Y.S., Jung S., Jang S., Kim Y.-K., Bai S.J., Park J.-H., Lee S.-K. (2017). Novel microbial photobioelectrochemical cell using an invasive ultramicroelectrode array and a microfluidic chamber. Biotechnol. Lett..

[18] Choi S. (2022). Electrogenic bacteria promise new opportunities for powering, sensing, and synthesizing. Small.

[19] Thakur A., Devi P. (2022). Paper-based flexible devices for energy harvesting, conversion and storage applications: A review. Nano Energy.

[20] Huang Q., Wang D., Zheng Z. (2016). Textile-based electrochemical energy storage devices. Adv. Energy Mater..

[21] Kuruvinashetti K., Tanneru H.K., Pakkiriswami S., Packirisamy M. (2023). Optical interactions in bio-electricity generation from photosynthesis in microfluidic micro-photosynthetic power cells. Energies.

[22] Muganli Z., Bütün I., Gharib G., Koşar A. (2024). Electricity generation using a microbial 3D bio-anode embedded bio-photovoltaic cell in a microfluidic chamber. Energy Adv..

[23] Park W.G., Kim M., Li S., Kim E., Park E.J., Yoo J., Maile N., Jae J., Kim H., Kim J.R. (2024). A light-driven photosynthetic microbial fuel cell for carbon-negative bioelectricity production. Sustain. Energy Fuels.

[24] Kwon Y., Hong D., Ahn Y. (2024). Monolayer textile-based co-laminar flow biocompatible enzymatic biofuel cell. Energy Conv. Manag..

[25] Kim J., Geon H., Ahn Y. (2024). Textile-based membraneless microfluidic double-inlet hybrid microbial-enzymatic biofuel cell. ACS Appl. Mater. Interfaces.

[26] Lee C.H., Ha H., Ahn Y., Liu H. (2023). Performance of single-layer paper-based co-laminar flow microbial fuel cells. J. Power Sources.

[27] Choban E.R., Waszczuk P., Kenis P.J.A. (2005). Characterizaion of limting factors in laminar flow-based membraneless microfuel cells. Electrochem. Solid State Lett..

[28] Liu L., Mohammadifar M., Elhadad A., Tahernia M., Zhang Y., Zhao W., Choi S. (2021). Spatial Engineering of Microbial Consortium for Long-Lasting, Self-Sustaining, and High-Power Generation in a Bacteria-Powered Biobattery. Adv. Energy Mater..

[29] Yang G., Choi S. (2018). Merging electric bacteria with paper. Adv. Mater. Technol..

